# Prevalence and Infection Load Dynamics of *Rickettsia felis* in Actively Feeding Cat Fleas

**DOI:** 10.1371/journal.pone.0002805

**Published:** 2008-07-30

**Authors:** Kathryn E. Reif, Rhett W. Stout, Gretchen C. Henry, Lane D. Foil, Kevin R. Macaluso

**Affiliations:** 1 Department of Pathobiological Sciences, Louisiana State University, Baton Rouge, Louisiana, United States of America; 2 Department of Biological Sciences, Louisiana State University, Baton Rouge, Louisiana, United States of America; The Scripps Research Institute, United States of America

## Abstract

**Background:**

*Rickettsia felis* is a flea-associated rickettsial pathogen recurrently identified in both colonized and wild-caught cat fleas, *Ctenocephalides felis*. We hypothesized that within colonized fleas, the intimate relationship between *R. felis* and *C. felis* allows for the coordination of rickettsial replication and metabolically active periods during flea bloodmeal acquisition and oogenesis.

**Methodology/Principal Findings:**

A quantitative real-time PCR assay was developed to quantify *R. felis* in actively feeding *R. felis*-infected fleas. In three separate trials, fleas were allowed to feed on cats, and a mean of 3.9×10^6^ *R. felis* 17-kDa gene copies was detected for each flea. A distinct *R. felis* infection pattern was not observed in fleas during nine consecutive days of bloodfeeding. However, an inverse correlation between the prevalence of *R. felis*-infection, which ranged from 96% in Trial 1 to 35% in Trial 3, and the *R. felis*-infection load in individual fleas was identified. Expression of *R. felis*-infection load as a ratio of *R. felis*/*C. felis* genes confirmed that fleas in Trial 3 had significantly greater rickettsial loads than those in Trial 1.

**Conclusion/Significance:**

Examining rickettsial infection dynamics in the flea vector will further elucidate the intimate relationship between *R. felis* and *C. felis*, and facilitate a more accurate understanding of the ecology and epidemiology of *R. felis* transmission in nature.

## Introduction

*Rickettsia felis* is an arthropod-associated intracellular, gram-negative bacterium with a worldwide distribution [1]. Molecular and serological evidence suggests *R. felis* is infectious for humans, causing a disease similar to murine typhus [2], although direct evidence of horizontal transmission of *R. felis* from arthropods to humans has not been demonstrated. Several studies have detected *R. felis* DNA in a diverse range of flea species of which *Ctenocephalides felis*, the cat flea, is considered the primary vector [3]. Laboratory studies have confirmed *R. felis* in *C. felis* populations is predominantly maintained by transstadial and transovarial transmission [4], [5]. Stable vertical transmission of *R. felis* has been examined in colonized fleas and the reported prevalence of *R. felis* in commercial and institutional *C. felis* colonies ranged from 43–100% [1], [5]–[7]. Within an isolated colony, such as the *C. felis* colony at Louisiana State University (LSU), prevalence of *R. felis* is variable with studies reporting a 65% and 100% prevalence of *R. felis* in 1999 [6] and 2007 [7], respectively. Vertical transmission of *R. felis* persists in *C. felis* for at least 12 generations without the aid of an *R. felis*-infected bloodmeal, however, over successive generations prevalence wanes to low levels (<10%) [5]. The specific mechanisms by which *R. felis* prevalence fluctuates within a *C. felis* colony are unknown, but in nature, the prevalence of *R. felis* in a flea population is likely amplified by fleas feeding on *R. felis*-infected mammalian hosts [8].

Ultrastructural analyses have been used to characterize *R. felis* within the flea host, as well as in cell lines [3], [9], [10]. Using electron microscopy, the presence of *R. felis* has been demonstrated in *C. felis* midgut epithelial cells, ovaries, salivary glands, and muscle [3], [10]. Additionally, molecular detection of *R. felis* has utilized traditional and quantitative real-time PCR (qPCR), typically targeting a portion of the genus-common 17-kDa antigen gene, for field sample diagnostics [7], [11], [12]. While qPCR has been utilized to assess the replication kinetics of *R. felis in vitro* [12], *in vivo* (arthropod and vertebrate) models of infection, for the purpose of examining the infection kinetics of *R. felis*, have not been examined. Development of such models will further elucidate the mechanisms for maintenance of *R. felis*, specifically in the flea populations that serve as vectors and reservoirs, and broaden our understanding of the ecology of *R. felis* transmission in nature.

Recent analysis of rickettsial replication in a *Rickettsia*/tick model (*Rickettsia amblyommii*/*Amblyomma americanum*) utilizing qPCR demonstrated a generalized dissemination of rickettsiae and, notably, a steady state level of rickettsial infection during relatively long (7–14 day) tick feeding periods [13]. While the direct regulators of rickettsial replication are under investigation in the slow-feeding tick host, the kinetics of rickettsial replication within the flea has not yet been examined. After locating a suitable host, cat fleas immediately begin feeding. Within 24 to 36 hr after initiating bloodfeeding, female cat fleas begin laying eggs and reach peak egg production (40 to 50 eggs/day) in 4 to 9 days [14]–[16]. Feeding adult fleas can live as long as 100 days, however, fleas that have initiated feeding and are removed from the host usually die within 24 to 48 hr [16]. Similar to other *Rickettsia*/arthropod relationships, *R. felis* is maintained via vertical transmission in the LSU colony of *C. felis*. We hypothesize that within colonized fleas, the intimate relationship between *R. felis* and *C. felis* allows for coordination of rickettsial replication and the metabolically active periods during flea bloodmeal acquisition and oogenesis. In the present study, the replication kinetics of *R. felis* in the cat-fed, LSU *C. felis* colony were investigated by developing a qPCR assay to detect and quantify *R. felis* in actively feeding cat fleas. Likewise, we considered the influence of flea gender and *R. felis* prevalence within the colony on rickettsial load in cat fleas during bloodmeal acquisition. A sensitive and specific method of detection, qPCR, allowed for accurate detection and quantification of *R. felis* in *C. felis*, including samples with low levels of infection.

## Materials and Methods

### Fleas and cats

Unfed *C. felis* were obtained from a colony maintained at LSU (Baton Rouge, LA). For the past 20 years, this colony has been persistently infected at a varying prevalence with *R. felis* [5]. At the Louisiana State University School of Veterinary Medicine (LSU-SVM) unfed newly-emerged, adult *C. felis* (mixed sex) were allowed to feed on 2 year-old, short-hair, flea-naïve, specific pathogen free cats (Harlan, Indianapolis, IN) as previously described by Foil et al. [17]. All cats were housed individually with 12∶12 light∶dark cycles at 20–22.2°C and 40–60% relative humidity. Pre-study blood collected from all cats was negative for *Rickettsia* by PCR. Briefly, fleas were contained in a capsule secured to the shaved lateral thorax of a cat and were able to feed through the fine mesh of the capsule. Before each trial (Day 0), colony *R. felis*-infection prevalence, infection load, and infection density of a minimum of nine individual fleas were assessed. Animal use in this research was conducted in accordance with protocols approved by the LSU Institutional Animal Care and Use Committee, and an approved protocol is on file in the office of the Division of Laboratory Animal Medicine at LSU-SVM.

### Experimental design

Three separate trials were conducted over the course of one year. Each trial was initiated approximately six months from the start of the previous trial. All fleas were drawn from the same colony, therefore, fleas in subsequent trials were descendants of the previous trial as the colony was not supplemented with outside fleas. The dynamic *R. felis*-infection prevalence in the LSU *C. felis* colony did not allow trials to be exact replicates of one another.

In each trial, capsules containing ∼100, unfed adult (mixed sex) fleas were secured to an individual cat. Approximately ten fleas were randomly selected and removed from the capsule for nine consecutive days. After collection, all flea samples were immediately frozen at −20°C until further processing. Every three days, fleas remaining in the study were transferred to a new capsule and returned to the cat.

### DNA Isolation

Fleas recovered daily from the cat host were individually sexed, assigned sample numbers, placed in microcentrifuge tubes and homogenized with sterile plastic pestles. Genomic DNA (gDNA) was extracted using either QIAGEN DNeasy Tissue Kit (Chatsworth, CA) according to the manufacturer's instructions and eluted in 50 µl Buffer AE (Trial 1), or using a modified version of the HotSHOT DNA extraction protocol (Trials 2 and 3) as described next [18]. Briefly, individual fleas were placed in microcentrifuge tubes partially submerged in liquid nitrogen and pulverized using sterile plastic pestles. Ground samples were incubated at 95°C for 45 min in 30 µl alkaline lysis reagent (25 mM NaOH, 0.2 mM disodium EDTA, pH of 12), cooled to 4°C for 5 min and mixed with 30 µl of neutralizing reagent (40 mM Tris-HCl, pH of 5). All gDNA preparations were stored at −20°C.

### Rickettsial Detection and Identification by PCR and Sequencing

*R. felis* infection in flea samples was detected by PCR amplification of a small portion of the *R. felis* 17-kDa antigen gene using primers (*Rf*17.153 5′-AGGACAGCTTGTCGGAGTAGG-3′ and *Rf*17.309 5′-ACGCCATTCTACGCTAGTGC-3′) derived from the complete genome of *R. felis* available on GenBank (Accession #: CP000053). A portion of *C. felis* 18S rDNA, amplified by primers (*Cf*18S 5′-TGCTCACCGTTTGACTTGG-3′ and *Cf*18S 5′-GTTTCTCAGGCTCCCTCTCC-3′) derived from an available partial sequence of *C. felis* 18S rDNA in GenBank (Accession #: AF136859), was used as a control to check the integrity of the template DNA. All primers used for standard PCR and qPCR were synthesized by Integrated DNA Technologies, Inc. (Coralville, IA). PCR products were amplified using isolated gDNA (individual flea lysates) or water (negative control) as template, gene-specific primers and 2× PCR Master Mix (Promega, Madison, WI). PCR conditions were as follows: initial denaturation at 94°C for 3 min, followed by 35 cycles of denaturation at 94°C for 30 s, annealing at 60°C for 45 s, extension at 72°C for 1 min, and a final extension at 72°C for 7 min. Amplified PCR products were visualized by electrophoresis on ethidium bromide stained 2.0% agarose gels. To verify identity, representative PCR products from each trial were cloned into pCR4-TOPO vectors (Invitrogen, Carlsbad, CA) and sequenced as previously described by Pornwiroon et al. [19].

### Southern Blot Analysis

A Southern blot was conducted to estimate the *C. felis* 18S rDNA copy number. Southern blot was performed as previously described by Horigane et al. [20]. Briefly, gDNA was extracted from *Rickettsia*-uninfected, unfed adult cat fleas (Heska Corporation, Loveland, CO) using Qiagen DNeasy DNA Extraction Kit and digested with five restriction enzymes (6 µg gDNA/enzyme): *Eag* I, *Pst I*, *Xba* I, *Xho* I (New England BioLabs, Ipswich, MA), and *Eco*R I (Promega, Madison, WI). DNA was separated on a 0.8% TAE gel and transferred to a positively charged nylon membrane (Amersham Biosciences, Piscataway, NJ) with 20× SSC, and fixed by UV. *C. felis* 18S rDNA was identified using the probe, *Cf*18SP, that was constructed based on a portion of the *C. felis* 18S rDNA sequence and amplified with primers *Cf* 18S_for_ and *Cf* 18S_rev_. The probe was labeled with PCR DIG Labeling Mix (Roche, Indianapolis, IN) according to the manufacturer's protocol. Anti-digoxigenin-AP (Fab fragment, Roche) and CDP Star (New England Bio Labs) were used for detection.

### Construction of Internal-Control Plasmid for Quantitative Real-Time PCR

To quantify copies of *R. felis* and *C. felis* genes in flea samples, serial dilutions of a plasmid containing single-copy portions of both the *R. felis* 17-kDa antigen gene and *C. felis* 18S rDNA were used to generate a standard curve. The plasmid was constructed by PCR amplification of a 157-base pair (bp) fragment of *R. felis* 17-kDa antigen gene and a 179-bp fragment of *C. felis* 18S rDNA with *Rf*17.153_for_-*Rf*17.309_rev_ and *Cf*18S_for_-*Cf*18S_rev_ primers, respectively. Each amplicon was cloned into to a pCR4-TOPO vector and sequenced to confirm identity as previously described [19]. Both amplicons were PCR amplified using a gene-specific primer and either a M13-forward or M13-reverse primer, digested with *Eco*R I and ligated together. The ligation product was amplified by PCR with primer pair *Rf*17.153_for_ and *Cf*18S_rev_, cloned and sequenced. The resulting plasmid, pCR4-TOPO-*Rf*17kDa+*Cf*18SrDNA, served as a standard template. The minimum detection limit for pCR4-TOPO-*Rf*17kDa+*Cf*18SrDNA was 10 copies.

### Quantitative Real-Time PCR

For each gene, qPCR components and template that included 2× iTaq SYBR Green Supermix (BioRad, Hercules, CA); 100 nM of each primer; DNase/RNase-free water; and 5 µl of gDNA template (samples), water (negative control), or serial 10-fold dilutions (1×10^8^ to 10 copies) of pCR4-TOPO-*Rf*17kDa+*Cf*18SrDNA were pre-mixed in 35 µl volumes in 96-well plates and aliquoted in triplicate 10 µl reactions on 384-well plates. The qPCR was performed with an ABI 7900HT unit (Applied Biosystems, Foster City, CA) at LSU-SVM using conditions previously described [13]. Results were analyzed with ABI 7900HT sequence detection system (SDS v2.3) software. The specificity of the assay was verified and the expected single peak for the internal-control plasmid and positive gDNA samples, but not in the water (negative control) samples, was identified in the dissociation curve. Additionally, representative qPCR products from each trial were verified by gel analysis to confirm the specificity of the reaction and cloned and sequenced to confirm identity (data not shown). Trial *R. felis*-infection prevalence in the *C. felis* colony was quantified as the percent of fleas positive for *R. felis* infection out of the total number examined per trial. *R. felis*-infection load was quantified as the copy number of *Rf*17kDa per individual flea lysate. *R. felis*-infection density was quantified as the ratio of log transformed *Rf*17kDa and log transformed *Cf*18S rDNA copy numbers (*Rf*17kDa/*Cf*18S) per individual flea.

### Statistical Analysis

Rickettsial load in fleas and the ratio of *Rf*17kDa/*Cf*18S were assessed after the logarithmic transformation of the quantity of the genes of interest (*Rf*17kDa and *Cf*18S). Analysis of variance, (SAS statistical package, Version 9.1.3, GLM procedure ANOVA, Cary, NC) was performed to examine potential differences between rickettsial load in fleas and ratio of *Rf*17kDa/*Cf*18S copy number over the study period; when overall significance was found, Tukey's honestly significant difference (HSD) post hoc test was used to examine pairwise differences of means of main effects. Pairwise *t*-tests of least square means were performed to determine any interaction effects between trial, gender, and experimental day for rickettsial infection load and ratio of *Rf*17kDa/*Cf*18S. An *F*-test was used for general comparisons of grouped means. For all comparisons, a *P*-value of <0.05 was considered significantly different.

## Results

### Prevalence of *R. felis* in LSU *C. felis* Colony

The specificity of *R. felis*-infection of *C. felis* (LSU colony) was confirmed by sequencing a portion of the 17-kDa antigen gene from a representative subset of fleas positive for *Rickettsia spp*. infection. Obtained sequences were compared to those in GenBank and all samples sequenced demonstrated a 100% identity to *R. felis* (accession number CP000053).

The prevalence of *R. felis*-infection in individual *C. felis* lysates was assessed by qPCR using primers that amplified a portion of the 17-kDa antigen gene. Trial prevalence and the daily prevalence range for each trial are listed in Table 1. *R. felis*-infection prevalence was greatest during Trial 1 and decreased 23% and 61% in Trials 2 and 3, respectively. When prevalence was assessed separately for male and female fleas, *R. felis* infection was gender independent.

**Table 1:** Trial *R. felis*-infection prevalence and daily range of cat fleas positive for *R. felis*-infection.

Trial	Trial prevalence	Daily range of cat fleas positive for *R. felis* infection
1	96%	89–100%
2	73%	40–90%
3	35%	10–60%
Mean of Trials	68%	46–83%

### Quantification of *Rf17*kDa and *Cf*18S Gene Copies During Flea Feeding

**Figure 1:**
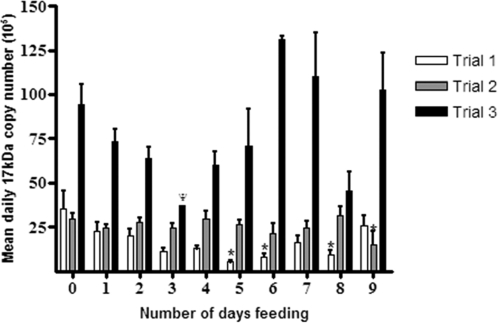
*R. felis*-infection load in individual *C. felis* lysates. For nine consecutive days, 10 fleas were randomly selected and removed from the feeding capsule situated on a cat host. In *Rickettsia* positive fleas, qPCR was used to quantify the mean (±SEM) *R. felis*-infection load. Results are presented as the mean daily *R. felis*-infection load (17-kDa antigen gene copy number) of individual, whole flea lysates during flea bloodmeal acquisition. Within each trial significant differences from Day 0 (unfed fleas) are marked with an asterisk. ^Ψ^In Trial 3, Day 3 only one flea was positive for *R. felis* infection.

To determine *R. felis*-infection load, fleas were individually titrated and the total number of *R. felis* (17-kDa copy number) in each individual flea sample was determined by qPCR. Serial dilutions of pCR4-TOPO-*Rf*17kDa+*Cf*18SrDNA were used to generate a standard curve and extrapolate *R. felis* quantities per individual flea lysate (Table S1a). The range of detected *Rf*17kDa was 3.1×10^1^ to 3.74×10^5^ copies per reaction, corresponding to a 1.3×10^3^ to 1.6×10^7^ total *Rf*17kDa load per flea lysate. The mean quantities of *R. felis* at each time point for individual flea samples in each trial are presented in Figure 1. Among all trials, fleas had significantly different *R. felis*-infection loads; increasing 4.75-fold during the course of the study between fleas in Trial 1 and Trial 3. Trial 3 fleas had the greatest *R. felis*-infection loads (mean load±SEM across all time points was 7.88×10^6^±5.43×10^5^) followed by Trial 2 (2.54×10^6^±1.40×10^5^) and Trial 1 (1.66×10^6^±1.62×10^5^). Combining data from all trials, when flea gender was considered, female fleas had significantly greater mean *R. felis*-infection loads than males. However, at individual time points within a trial or across trials there were few significant differences in *R. felis*-infection loads between male and female fleas.

When the mean daily *R. felis*-infection loads for each trial, and for all trials combined, were compared, significant variability in *R. felis*-infection load was observed by day compared to the starting point at Day 0 within Trials 1 and 2 (Figure 1). No significant variability in daily *R. felis*-infection load was observed in Trial 3, when compared to the Day 0 infection load. Analyses of the mean daily *R. felis* infection load combining all Trials demonstrated that there was no single day when *R. felis*-infection load was consistently greater within the study period. Likewise, although variability in *R. felis*-infection load was observed in some trials, there was no consistent pattern of increased or decreased rickettsial infection across all trials. Therefore, a consistent *R. felis*-infection load during flea feeding was observed as there was no significant correlation between *R. felis* replication and flea feeding or oviposition events.

To estimate flea sample size, *R. felis*-infected and -uninfected *C. felis* were individually titrated and the *Cf*18S rDNA copy number within a single flea was determined by qPCR. Serial dilutions of pCR4-TOPO-*Rf*17kDa+*Cf*18SrDNA were used to generate a standard curve and extrapolate *Cf*18S rDNA copy number per individual *C. felis* lysate (Table S1b). Within individual trials, mean *Cf*18S copy numbers did significantly vary during the feeding period on some days, however there was no single day where *Cf*18S copy numbers were consistently different. For both *R. felis*-infected and -uninfected fleas, the mean *Cf*18S copy numbers increased with each subsequent Trial, with a significant 2.6-fold increase in mean *Cf*18S copy numbers between Trials 2 and 3. When data from all three trials were combined, significantly greater *Cf*18S copy numbers were observed in female *C. felis* compared to male *C. felis*. Likewise, uninfected *C. felis* had significantly greater mean *Cf*18S copy numbers compared to *R. felis*-infected *C. felis*.

### *R. felis*-infection Load in *C. felis* Expressed as a Ratio of *Rf*17kDa/*Cf*18S

**Figure 2:**
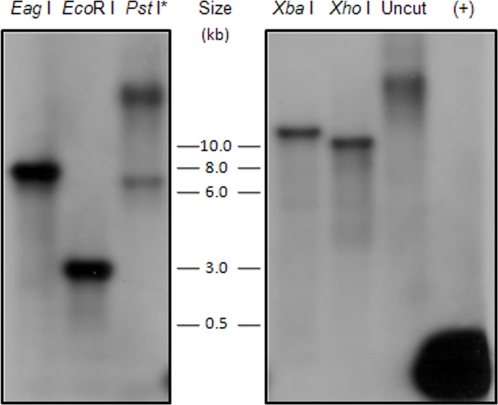
Estimation of *C. felis* 18S rDNA copy number using Southern blot. *C. felis* genomic DNA (6 µg/enzyme) was digested with *Eag* I, *Eco*R I, *Pst* I, *Xba* I and *Xho* I. Uncut gDNA served as a negative control and PCR product of a portion of *C. felis* 18S rDNA served as a positive control. Genomic DNA was hybridized with *Cf*18S bp probe to estimate the number of 18S rDNA gene copies in *C. felis*. Digestion with each enzyme results in a single digestion product. (*DNA was not completely digested by *Pst* I. Top band is uncut DNA.)

*R. felis*-infection of *C. felis* was further assessed as a ratio of *R. felis* and *C. felis* genes. Examination of the published genome demonstrated that the *R. felis* 17-kDa antigen gene is a single-copy gene [21]. In this study, the copy number of *C. felis* 18S rDNA was estimated by Southern blot analysis. *C. felis* gDNA was digested with 5 restriction enzymes all resulting in the presence of a single band of variable size (dependant on enzyme used) (Figure 2). Although each enzyme produced a single band indicating that the *C. felis* 18S rDNA gene may be single-copy, a tandem arrangement of multiple gene copies may not be discernable using Southern blot analysis alone.

**Figure 3:**
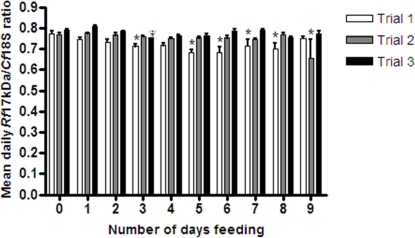
*R. felis*-infection load in *C. felis* expressed as a ratio of *R. felis* and *C. felis* genes. For nine consecutive days 10 fleas were randomly selected and removed from the feeding capsule situated on a cat host. In *Rickettsia* positive fleas, *R. felis* infection was determined by quantifying the *R. felis* 17-kDa copy number. For *R. felis*-infected fleas, *C. felis* 18S rDNA copy number was quantified to serve as a comparison point for *R. felis* infection. *R. felis*-infection density was determined by logarithmically transforming and taking the ratio of *R. felis* 17-kDa and *C. felis* 18S rDNA (*Rf*17kDa/*Cf*18S) copy numbers and the mean (±SEM) daily *Rf*17kDa/*Cf*18S ratios for each trial were calculated. Within each trial significant differences from Day 0 (unfed fleas) are marked with an asterisk. ^Ψ^In Trial 3, Day 3 only one flea was positive for *R. felis* infection. All trials have significantly higher ratios than the previous trial, indicating that rickettsial burdens are increasing in the LSU *C. felis* colony.

For individual flea lysates positive for *R. felis*, *R. felis*-infection density was examined. Assessed by qPCR, copies of *Rf*17kDa and *Cf*18S were determined relative to their positions in the standard curve and extrapolated to the individual flea lysate. To determine *R. felis*-infection density *Rf*17kDa/*Cf*18S ratios for individual flea samples were calculated after the logarithmic transformation of the quantity of the genes of interest (*Rf*17kDa and *Cf*18S). The mean daily *Rf*17kDa/*Cf*18S ratios for each trial are presented in Figure 3.

The mean (±SEM) trial *Rf*17kDa/*Cf*18S ratio increased significantly between subsequent trials from 0.72±0.0064 to 0.75±0.0059 to 0.78±0.0032, in Trials 1–3, respectively. When flea gender was considered, female fleas in Trials 2 and 3 had significantly greater *Rf*17kDa/*Cf*18S ratios than female fleas in Trial 1, but were not significantly different from one another. Male fleas in Trials 1 and 2 did not have significantly different *Rf*17kDa/*Cf*18S ratios from one another, but both had significantly lower ratios than male fleas in Trial 3. Comparing female and male fleas within trials, males had significantly greater *Rf*17kDa/*Cf*18S ratios in Trial 1 than females, but in Trial 2 females had significantly greater *Rf*17kDa/*Cf*18S ratios than males. Due to the limited occurrence of *R. felis* infection in male fleas in Trial 3, gender-based ratio differences could not be statistically assessed.

In individual trials, significant differences in *Rf*17kDa/*Cf*18S ratios were observed on multiple days, however, among all trials no single day was consistently significantly different from Day 0. The mean daily *Rf*17kDa/*Cf*18S ratios for all trials were combined and compared, independent of flea gender, and no significant variability in *Rf*17kDa/*Cf*18S ratio was observed by day compared to the starting point at Day 0. Analyses of the mean daily *Rf*17kDa/*Cf*18S ratio for female fleas, combining and comparing all trials, identified a significantly lower ratio on Day 6 compared to Day 0, a trend similar to that observed for females in Trial 1, but not females in Trials 2 and 3. No significant differences in mean daily *Rf*17kDa/*Cf*18S ratios for all trials combined were observed for male fleas on any day compared. Additionally, a correlation between the ratio of *Rf*17kDa/*Cf*18S and flea bloodmeal acquisition was not observed. For female fleas, during the time of peak oviposition (Day 6) there was a decrease in *Rf*17kDa/*Cf*18S ratio. No distinct relationship was observed between *R. felis*-infection load and *C. felis* 18S copy number. A consistent infection rate of *R. felis*, with minimal significant increases or decreases in infection load is maintained during flea metabolically active periods.

## Discussion

Both traditional and real-time PCR have been used to further characterize rickettsial infection in fleas. Amplification of a portion of the gene encoding the rickettsial-common 17-kDa antigen allowed for identification and subsequent differentiation by restriction fragment length polymorphism of both *Rickettsia typhi* and *R. felis* in *C. felis* [22]. In this study, the presence of *R. felis* as the rickettsial species infecting the LSU *C. felis* flea colony was confirmed by traditional PCR and gene sequence analysis. Real-time PCR has also been used for quantitative assessment of *R. felis* replication and as a diagnostic tool to assess *R. felis* infection in arthropod hosts. Replication of four isolates of *R. felis*, including the LSU strain, was measured in XTC-2 cells treated with antibiotics; however, the techniques for establishing rickettsial gene copy numbers were not provided and the qPCR assay utilized is not clear [12]. More recently, a probe-based qPCR targeting rickettsial *ompB* for detection and differentiation of *R. felis* and *R. typhi* in colonized and wild-caught *C. felis* was described [7]. It was demonstrated that crude extraction (boiled flea lysate) of DNA was an insensitive procedure, compared to kit-based DNA extraction, resulting in limited detection of *Rickettsia*. In the present report, we utilized qPCR to analyze the rickettsial load in individual *C. felis* during flea feeding and oviposition. Differences in sensitivity between kit-based and HotSHOT DNA recovery techniques utilized in the current study were undetectable. Also, Henry *et al* [7] utilized serial dilutions of a plasmid containing a portion of the *ompB* target sequence and determined the sensitivity of the assay to be 1 copy/µl; 10 times greater sensitivity than the 17-kDa antigen gene target sequence and SYBR Green assay in the current study. Although there is increased sensitivity in the probe-based assay compared to our SYBR Green assay, the large rickettsial load in LSU fleas does not require detection of low numbers of *R. felis*. Examining gene copy numbers by qPCR is limited in differentiating between live/dead organisms; however, DNA analysis can still provide a reasonable assessment of infection load in hosts that are naturally infected and/or utilized in the vertical transmission of the organism. This study provides the first application of qPCR to examine the kinetics of *R. felis* infection within its anautogenous vector and primary reservoir, the cat flea.

Wide dissemination of vertically maintained bacteria is common in arthropods [23], [24]. Within the flea, *R. feli*s infects many types of tissues, including the salivary glands [3], [10]; in this study, *R. felis* infection load was examined at the whole individual flea level. Among the *R. felis*-infected fleas, we did identify a mean of 3.9×10^6^ rickettsiae during feeding. All trials were significantly different in their mean *R. felis* infection load, with Trial 1 fleas and Trial 3 fleas having the lowest and highest individual flea rickettsial loads, respectively. Regardless of whether infection load was quantified as total load per flea or as a ratio of *R. felis* and *C. felis* genes, rickettsial load was not definitively affected by flea bloodmeal acquisition or oogenesis, nor was there a consistent *R. felis* replication pattern observed across all trials. The significant decrease in the mean trial *Rf*17kDa/*Cf*18S ratio on Day 6 is skewed by the results of Trial 1, in which more fleas were positive for *R. felis*-infection. We have utilized qPCR to examine spotted fever group rickettsiae within the tick *A. americanum* and, similar to this study, we identified a steady level of *R. amblyommii* load associated with bloodmeal acquisition when infection was assessed on an individual tissue basis [13]. Therefore, in the two models examined, there appears to be a balance between rickettsial load and host size in actively feeding arthropods. Future studies examining *R. felis* infection within its flea vector and determination of infection density within specific tissues (*e.g.* salivary glands and ovaries) will further elucidate the biological interactions between rickettsiae and flea hosts.

In addition to *C. felis*, *R. felis* has been molecularly identified in numerous species of wild-caught fleas, including *C. canis* [25], *Xenopsylla cheopis* [26], *Archaeopsylla erinacei* [27], *Spilopsyllus cuniculi* [28], *Echidnophaga gallinacean* [28], *Anomiopsylla nudata* [11]; and, while it is likely that the prevalence of *R. felis* in a flea population is amplified by an infectious bloodmeal, the acquisition mechanisms and stability of transmission are not known. The prevalence of *R. felis* in wild-caught fleas has been reported to be 1–20%, which is typically lower than that observed in colonized fleas [1], [29]–[32]. The efficient replication and vertical transmission of *R. felis* within fleas may minimize the necessity of frequent mammalian infections to maintain *R. felis* in nature; however, experimental evidence indicates the transmission cycle of *R. felis* is more complex. In laboratory reared flea colonies, such as the LSU *C. felis* colony, the presence and dynamic prevalence of *R. felis* make it a valuable tool to examine *Rickettsia*/flea interactions [19], [32]. Wedincamp and Foil [5] investigated the efficiency of *R. felis* vertical transmission in *C. felis* without the aid of an infectious bloodmeal and demonstrated fluctuating, but decreasing prevalence through twelve generations. During the last 15 years, several independent studies have identified the variable prevalence of *R. felis*, ranging from 43–100% [7], [19], [22], [33]. The LSU *C. felis* colony is maintained solely on cat hosts, but the role of cat hosts as a source of infectious bloodmeal is unclear. Cats continuously fed on by *R. felis*-infected fleas seroconvert two to four months post-exposure [6]. *R. felis* DNA has been detected in cat blood [6]; however, recovery of *R. felis* from these cats has been unsuccessful thus far [K. R. Macaluso, unpublished data]. Each trial utilized a flea-naïve cat host, minimizing the possibility of cat-derived, immune-mediated rickettsial clearance in feeding fleas. Interestingly, even when fleas are fed only on live hosts, there is variance in *R. felis* prevalence between generations as demonstrated in the current study that utilized subsequent generations of fleas for each trial. After a substantial population loss followed by a population expansion in the LSU *C. felis* colony, the prevalence of *R. felis* in the flea population neared 100% [19]. In individual fleas, 3.1×10^1^ to 3.74×10^5^ of *Rf*17kDa copies per reaction were observed, with only four fleas having detectable *Rf*17kDa copy numbers (in total lysate) under 1.0×10^4^. Although not proven in this study, the dramatic contrast of *Rf*17kDa copy numbers of these four fleas in comparison to the remaining *R. felis*-infected fleas may suggest a role for low-level horizontal acquisition of *R. felis* in these few fleas from feeding on a shared host or larval cannibalism. Under laboratory conditions with either natural or artificial hosts, the mechanisms of prevalence and infection load fluctuations are intriguing and require further study to assess if carriage of *R. felis* is beneficial to *C. felis*.

Alternatively, there may be microbial-dependent influence on *R. felis* prevalence, as we have identified intracellular *Wolbachia* spp. in the fleas [19]. *Wolbachia* spp. infect many arthropods and are readily able to manipulate their arthropod host (*e.g.* feminization, cytoplasmic incompatibility) with a potential to impact host fitness [23], [34], [35]. While *Wolbachia* spp. have been identified in fleas, their impact on flea fitness and relationship with other flea microbiota, such as *R. felis*, have not been examined [19], [36]. Not only can microbiota compete with one another for host resources, they can often manipulate their environment affecting arthropod-host fitness [34], [35]. For example, vertical transmission of the tick-borne human rickettsial pathogens, *R. rickettsii*, *R. parkeri*, and *R. conorii*, are associated with decreased arthropod-host fitness, whereas vertical maintenance of other *Rickettsia* spp. do not impact tick fitness [13], [37], [38]. Interaction and possible competition of vertically transmitted microbiota and their potential impact on host fitness is likely complex and needs to be scrutinized. Utilization of qPCR and laboratory models of *R. felis* transmission will further elucidate mechanisms of transmission in nature.

**Figure 4:**
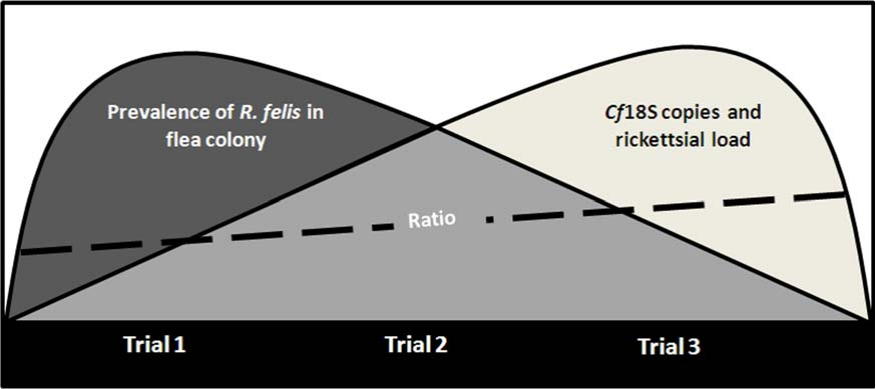
Experimental model depicting the relationship between *R. felis* and *C. felis*. Supported by the results of this study, *R. felis* prevalence and individual flea infection load in the LSU *C. felis* colony are inversely correlated. The ratio of *Rf*17kDa/*Cf*18SrDNA also increased significantly between Trial 1 and Trial 3 indicating fleas are infected at a greater density. Trials are situated within the model according to their individual results. As a population, fleas in Trial 1 had the highest prevalence of *R. felis* infection and the lowest mean individual *R. felis*-infection load. Conversely, fleas in Trial 3 had the lowest prevalence of *R. felis*-infection and the greatest mean individual *R. felis*-infection load. Trial 2 fleas represent a median demonstrating the progression of decreasing colony prevalence and increasing infection load from Trial 1 to Trial 3. The ratio of *R. felis* and *C. felis* genes increases significantly from Trial 1 to Trial 3, demonstrating that fleas in Trial 3 have a greater *R. felis* burden (higher *Rf*17kDa/*Cf*18S ratio) than fleas in Trial 1. These results indicate that at increased infection loads, *R. felis* may influence flea fitness to facilitate their own successful transmission to the next generation of fleas or to a susceptible mammalian host.

Although no clear replication pattern was observed in actively bloodfeeding and ovipositing fleas, there was an inverse correlation between colony *R. felis*-infection prevalence and *R. felis*-infection load in individual fleas. Specifically, as the prevalence of *R. felis* decreased in our flea colony from 96% in Trial 1 to 35% in Trial 3, the mean *R. felis*-infection load in individual fleas increased 4.75-fold (Figure 4). Quantification of increasing *R. felis*-infection was demonstrated at the whole flea level (counting *Rf*17kDa copy numbers) and verified again when assessing infection as a ratio of *Rickettsia* and flea genes. *C. felis* 18S rDNA copies also increased in both *R. felis*-infected and uninfected fleas across all trials. The ratio of *R. felis* and *C. felis* genes increases significantly from Trial 1 to Trial 3, demonstrating that the larger fleas (greater *Cf*18S quantities) in Trial 3 have an unproportionately greater *R. felis* burden than the smaller fleas in Trial 1. These results verify our *R. felis*-infection load per individual flea lysate findings, supporting that infection load is increasing beyond the expected proportional increase relative to *C. felis* 18S rDNA copy number. Whether or not larger, more densely infected fleas are more competent vectors for *R. felis* will be interesting to explore further.

Variable prevalence and infection density in *C. felis* may represent a *R. felis* ecological maintenance strategy, in which waning prevalence signals, by an unknown mechanism, increased infection burdens in individual fleas potentially facilitating more efficient transmission to progeny (vertical transmission) or a reservoir host (horizontal transmission) in order to persist in the flea population. Here we describe the first quantitative assessment of *R. felis* infection in fleas. We also have data to suggest that the previously unrecognized horizontal transmission of *R. felis* occurs among fleas and that prevalence of *R. felis* in the population is correlated to individual flea rickettsial load. The results of this study will help elucidate the epidemiology of *R. felis*, an emerging infectious pathogen, by demonstrating the dynamic prevalence and infection density changes that occur within a flea population.

## Supporting Information

Table S1 Raw data for gene copy numbers of (a) *R. felis* 17-kDa and (b) *C. felis* 18S rDNA in individual flea samples. In three independent trials spanning one year, individual fleas were assessed by qPCR for *R. felis* infection prior to the start of the feeding (Day 0) and during nine days of feeding on cat hosts. (a) *R. felis* 17-kDa gene copy number quantities per individual flea lysate were extrapolated based on their position relative to the standard curve and listed in the above table. Individual fleas negative for infection with *R. felis* are represented by ‘-’. (b) *C. felis* 18S rDNA gene copy number quantities per individual flea lysate were also determined by extrapolating their values based on their position relative to the standard curve and are listed in the above table. *C. felis* 18S rDNA counts were performed for all fleas, however, fleas determined to be infected with *R. felis* are shaded in gray. The values in the above tables were log transformed and ratios of log*Rf*17kDa/log*Cf*18SrDNA were generated for individual fleas samples (results presented in main text).
(0.04 MB PDF)
