# Supplementary material for: Prevalence and Infection Load Dynamics of Rickettsia felis in Actively Feeding Cat Fleas
Source: PLoS One. 2008 Jul 30;3(7):e2805. doi: 10.1371/journal.pone.0002805 (PMC2474969; doi:10.1371/journal.pone.0002805)
Supplement: Table S1 — Raw data for gene copy numbers of (a) R. felis 17-kDa and (b) C. felis 18S rDNA in individual flea samples. In three independent trials spanning one year, individual fleas were assessed by qPCR for R. felis infection prior to the start of the feeding (Day 0) and during nine days of feeding on cat hosts. (a) R. felis 17-kDa gene copy number quantities per individual flea lysate were extrapolated based on their position relative to the standard curve and listed in the above table. Individual fleas negative for infection with R. felis are represented by ‘-’. (b) C. felis 18S rDNA gene copy number quantities per individual flea lysate were also determined by extrapolating their values based on their position relative to the standard curve and are listed in the above table. C. felis 18S rDNA counts were performed for all fleas, however, fleas determined to be infected with R. felis are shaded in gray. The values in the above tables were log transformed and ratios of logRf17kDa/logCf18SrDNA were generated for individual fleas samples (results presented in main text). (0.04 MB PDF) [file pone.0002805.s001.pdf]

Table S1a. Copy number of *Rf* 17kDa in individual flea lysates

	Exp. day	Total no. of fleas surveyed (no. *R.felis*- infected/no. uninfected)	*Rf* 17kDa counts in individual flea lysate samples										Daily mean ±SEM *R. felis*-infection load
			1	2	3	4	5	6	7	8	9	10	
**Trial 1**	**0**	9 (8/1)	4.79E+05	5.85E+06	2.47E+06	9.20E+06	1.97E+06	1.60E+06	1.45E+06	-	5.20E+06		3.53E+06 ± 1.05E+06
	**1**	10 (10/0)	2.41E+06	3.86E+06	7.11E+05	1.88E+06	9.93E+05	4.69E+06	4.76E+06	2.94E+05	2.08E+06	7.97E+05	2.25E+06 ± 5.26E+05
	**2**	10 (10/0)	3.21E+06	2.40E+06	1.28E+06	1.62E+06	7.01E+05	8.42E+05	4.40E+06	1.52E+06	2.61E+06	1.69E+06	2.03E+06 ± 3.62E+05
	**3**	10 (9/1)	9.58E+05	1.73E+06	4.94E+05	1.81E+05	1.79E+06	2.11E+06	1.07E+06	7.33E+05	-	7.61E+05	1.09E+06 ± 2.17E+05
	**4**	10 (9/1)	3.65E+05	1.84E+06	1.82E+06	6.10E+05	-	8.20E+05	1.82E+06	1.34E+06	1.31E+06	1.54E+06	1.27E+06 ± 1.85E+05
	**5**	10 (9/1)	4.75E+05	6.05E+05	1.05E+06	-	3.31E+05	8.55E+05	8.10E+05	2.51E+05	6.86E+04	1.32E+05	5.08E+06 ± 1.14E+05
	**6**	10 (10/0)	1.49E+05	1.77E+05	1.07E+05	9.49E+05	1.97E+06	1.11E+06	1.31E+06	1.52E+06	7.00E+05	4.04E+03	7.99E+05 ± 2.16E+05
	**7**	10 (10/0)	2.15E+06	7.82E+05	1.04E+06	3.10E+05	2.05E+06	2.28E+06	4.94E+03	1.06E+06	3.01E+06	3.79E+06	1.65E+06 ± 3.83E+05
	**8**	10 (10/0)	3.26E+05	5.83E+04	1.93E+06	1.28E+06	1.77E+04	6.12E+05	1.67E+05	1.40E+06	2.19E+06	1.10E+06	9.08E+05 ± 2.49E+05
	**9**	10 (10/0)	7.19E+05	1.58E+06	4.56E+05	1.79E+06	2.94E+06	5.08E+06	6.11E+06	1.76E+06	3.94E+06	1.12E+06	2.55E+06 ± 6.06E+05
												**Mean *R. felis* -Infection Load**	**1.66E+06 ± 1.62E+05**
**Trial 2**	**0**	40 (35/5)	2.29E+06	2.35E+06	2.76E+06	2.25E+06	1.16E+06	2.45E+06	2.53E+06	2.74E+06	1.45E+06	3.48E+06	2.98E+06 ± 3.33E+05
			2.34E+06	2.59E+06	1.42E+06	1.58E+06	2.18E+06	1.28E+06	3.02E+06	-	3.13E+06	2.25E+06	
			-	3.00E+06	1.84E+06	2.79E+06	2.90E+06	1.33E+06	3.17E+06	-	2.24E+06	3.62E+06	
			3.35E+06	8.67E+06	5.59E+06	2.90E+06	6.08E+06	-	1.30E+03	-	6.62E+06	6.99E+06	
	**1**	10 (8/2)	2.58E+06	1.36E+06	1.78E+06	2.90E+06	2.79E+06	3.38E+06	2.27E+06	-	2.37E+06	-	2.43E+06 ± 2.26E+05
	**2**	10 (6/4)	2.97E+06	-	3.01E+06	1.64E+06	2.99E+06	2.83E+06	-	3.15E+06	-	-	2.76E+06 ± 2.29E+05
	**3**	12 (10/2)	-	2.28E+06	1.38E+06	1.39E+06	2.01E+06	1.98E+06	-	2.67E+06	4.67E+06	2.60E+06	2.43E+06 ± 2.95E+05
			2.52E+06	2.79E+06									
	**4**	10 (6/4)	1.59E+06	3.64E+06	-	2.81E+06	1.70E+06	4.40E+06	3.45E+06	-	-	-	2.93E+06 ± 4.57E+05
	**5**	10 (9/1)	2.21E+06	2.58E+06	3.40E+06	2.82E+06	3.24E+06	-	2.52E+06	8.78E+05	3.43E+06	2.61E+06	2.63E+06 ± 2.61E+05
	**6**	10 (6/4)	4.67E+06	-	2.34E+06	-	9.58E+05	-	1.23E+06	2.60E+06	-	1.03E+06	2.14E+06 ± 5.80E+05
	**7**	10 (5/5)	-	2.01E+06	2.80E+06	-	-	-	1.36E+06	2.38E+06	-	3.67E+06	2.44E+06 ± 3.86E+05
	**8**	10 (8/2)	1.93E+06	4.33E+06	-	5.27E+06	2.33E+06	4.55E+06	1.94E+06	-	1.35E+06	3.60E+06	3.16E+06 ± 5.16E+05
	**9**	10 (4/6)	2.59E+06	-	2.97E+06	-	-	-	-	3.14E+05	-	2.32E+03	1.47E+06 ± 7.64E+05
												**Mean *R. felis* -Infection Load**	**2.54E+06 ± 1.40E+05**
**Trial 3**	**0**	27 (9/18)	-	-	-	-	-	-	-	1.03E+07	1.10E+07	1.44E+07	9.43E+06 ± 1.12E+06
			-	-	1.23E+07	-	-	1.05E+07	1.03E+07	-	-	-	
			-	-	-	4.23E+06	5.21E+06	6.61E+06	-				
	**1**	10 (5/5)	6.72E+06	-	9.19E+06	5.86E+06	-	8.94E+06	-	5.86E+06	-	-	7.31E+06 ± 7.33E+05
	**2**	10 (5/5)	5.53E+06	-	5.14E+06	5.32E+06	-	-	-	-	8.19E+06	7.72E+06	6.38E+06 ± 6.51E+05
	**3**	10 (1/9)	3.68E+06	-	-	-	-	-	-	-	-	-	3.68E+06
	**4**	10 (6/4)	3.65E+06	4.32E+06	9.11E+06	6.33E+06	6.22E+06	6.44E+06	-	-	-	-	6.01E+06 ± 7.81E+05
	**5**	10 (4/6)	-	-	1.01E+07	1.11E+07	-	-	-	-	2.57E+06	4.55E+06	7.09E+06 ± 4.17E+06
	**6**	10 (2/8)	-	-	1.33E+07	-	-	1.29E+07	-	-	-	-	1.31E+07 ± 2.18E+05
	**7**	10 (4/6)	-	-	-	-	8.36E+06	1.57E+07	-	1.46E+07	5.28E+06	-	1.10E+07 ± 2.50E+06
	**8**	10 (3/7)	-	-	-	-	4.64E+06	-	-	-	2.69E+06	6.32E+06	4.55E+06 ± 1.05E+06
	**9**	10 (2/8)	-	1.23E+07	-	8.16E+06	-	-	-	-	-	-	1.02E+07 ± 2.08E+06
												**Mean *R. felis* -Infection Load**	**7.88E+06 ± 5.43E+05**

Table S1b. Copy number of *Cf* 18S in individual flea lysates

	Exp. Day	Total no. of fleas surveyed (no. *R.felis*- infected/no. uninfected)	*Cf* 18S count for individual flea lysate samples										Daily mean ±SEM *Cf* 18S count for *R. felis* -infected fleas	Daily mean ±SEM *Cf* 18S count for uninfected fleas
			1	2	3	4	5	6	7	8	9	10		
**Trial 1**	**0**	9 (8/1)	5.28E+07	2.21E+08	2.90E+08	3.98E+08	2.65E+08	3.34E+08	1.53E+08	1.41E+08	1.36E+08		2.31E+08 ± 4.01E+07	1.41E+08
	**1**	10 (10/0)	5.52E+08	2.72E+08	1.16E+08	6.87E+07	9.97E+07	6.45E+08	8.39E+08	7.71E+07	4.25E+08	1.59E+08	3.25E+08 ± 2.75E+07	n/a
	**2**	10 (10/0)	8.56E+08	4.07E+08	4.80E+08	2.82E+08	3.78E+08	1.24E+08	1.25E+08	2.79E+08	3.80E+08	5.97E+08	3.91E+08 ± 2.19E+07	n/a
	**3**	10 (9/1)	3.60E+08	2.03E+08	1.12E+08	2.59E+07	1.73E+08	8.68E+08	9.31E+08	1.61E+08	4.80E+08	4.16E+08	3.61E+08 ± 1.09E+08	4.80E+08
	**4**	10 (9/1)	2.17E+08	3.65E+08	6.40E+08	4.24E+08	3.01E+08	8.80E+08	5.64E+08	8.76E+07	1.38E+08	1.37E+08	3.84E+08 ± 8.99E+07	3.01E+08
	**5**	10 (9/1)	8.55E+08	1.93E+08	1.94E+08	2.56E+07	3.87E+07	2.05E+08	3.08E+08	2.80E+08	2.26E+07	6.93E+07	2.41E+08 ± 8.39E+07	2.56E+07
	**6**	10 (10/0)	2.72E+07	1.01E+08	5.21E+07	2.72E+08	4.83E+08	1.06E+08	2.82E+08	1.99E+08	1.21E+08	2.14E+08	1.86E+08 ± 4.30E+07	n/a
	**7**	10 (10/0)	3.33E+08	9.23E+07	5.60E+07	5.34E+07	1.56E+08	2.71E+08	2.11E+08	3.08E+08	5.26E+08	8.91E+08	2.90E+08 ± 8.12E+07	n/a
	**8**	10 (10/0)	7.59E+07	2.57E+08	2.59E+08	2.34E+08	1.22E+08	1.07E+08	2.12E+07	4.65E+07	2.84E+08	1.90E+08	1.60E+08 ± 3.06E+07	n/a
	**9**	10 (10/0)	1.01E+08	1.46E+08	1.02E+08	2.07E+08	2.16E+08	3.61E+08	7.33E+08	3.73E+08	4.26E+08	1.31E+08	2.80E+08 ± 6.28E+07	n/a
												**Mean *Cf* 18S Count**	**2.85E+08 ± 2.35E+07**	**2.37E+08 ± 9.89E+07**
**Trial 2**	**0**	40 (35/5)	2.25E+08	1.10E+08	2.27E+08	2.40E+08	9.02E+07	1.97E+08	2.56E+08	4.41E+08	1.16E+08	4.44E+08	3.94E+08 ± 8.46E+07	5.03E+08 ± 2.52E08
			6.49E+07	2.00E+08	1.03E+08	1.42E+08	7.82E+07	1.22E+08	2.37E+08	3.70E+08	3.01E+08	1.80E+08		
			8.39E+07	2.50E+08	6.42E+07	3.09E+08	3.90E+08	9.02E+07	5.02E+08	1.13E+08	2.33E+08	3.40E+08		
			1.98E+08	1.76E+09	1.22E+09	7.32E+08	2.54E+09	4.81E+08	4.04E+08	1.47E+09	5.28E+08	4.40E+08		
	**1**	10 (8/2)	1.71E+08	6.92E+07	1.13E+08	2.13E+08	2.72E+08	2.31E+08	1.92E+08	1.38E+08	1.82E+08	1.88E+08	2.90E+08 ± 8.12E+07	1.63E+08 ±2.49E+07
	**2**	10 (6/4)	1.78E+08	1.96E+08	1.20E+08	2.68E+08	4.65E+08	3.88E+08	1.70E+08	2.61E+08	2.57E+08	3.41E+08	2.90E+08 ± 8.12E+07	2.41E+08 ± 3.79E+08
	**3**	12 (10/2)	1.39E+08	2.47E+08	1.30E+08	1.15E+08	2.34E+08	1.38E+08	4.11E+08	3.35E+08	6.08E+08	1.84E+08	2.91E+08 ± 5.76E+07	2.75E+08 ± 1.36E+08
			6.04E+08	3.17E+08										
	**4**	10 (6/4)	3.84E+08	4.12E+08	1.67E+08	5.13E+08	2.50E+08	3.94E+08	4.77E+08	3.29E+08	3.56E+08	4.22E+08	4.5E+08 ± 3.72E+07	3.18E+08 ± 5.42E+07
	**5**	10 (9/1)	6.94E+08	4.27E+08	2.92E+08	3.81E+08	2.33E+08	2.05E+08	3.59E+08	1.14E+08	3.64E+08	3.33E+08	3.55E+08 ± 5.25E+07	2.05E+08
	**6**	10 (6/4)	4.40E+08	4.18E+08	1.48E+08	1.71E+08	1.83E+08	1.69E+08	1.35E+08	2.79E+08	1.49E+08	1.81E+08	2.28E+08 ± 4.72E+07	2.27E+08 ± 6.39E+07
	**7**	10 (5/5)	3.22E+08	3.81E+08	5.31E+08	1.47E+08	1.87E+08	2.16E+08	1.77E+08	3.20E+08	7.82E+08	4.41E+08	3.70E+08 ± 5.95E+07	3.31E+08 ± 1.16E+08
	**8**	10 (8/2)	3.77E+08	5.68E+08	2.33E+08	3.57E+08	1.66E+08	2.57E+08	3.21E+08	6.60E+07	1.12E+08	1.20E+08	2.85E+08 ± 5.46E+07	1.50E+08 ± 8.37E+07
	**9**	10 (4/6)	1.96E+08	2.65E+07	2.36E+08	4.52E+08	2.05E+08	2.40E+08	2.01E+08	1.42E+08	3.25E+08	2.01E+08	1.94E+08 ± 1.94E+07	2.42E+08 ± 5.79E+07
												**Mean *Cf* 18S Count**	**2.98E+08 ± 3.29E+07**	**2.65E+08 ± 4.18E+07**
**Trial 3**	**0**	27 (9/18)	1.12E+09	7.00E+08	7.14E+08	8.28E+08	1.04E+09	9.31E+08	5.17E+08	8.51E+08	5.57E+08	1.16E+09	6.57E+08 ± 9.01E+07	7.66E+08 ± 7.29E+07
			8.78E+08	1.29E+09	6.74E+08	8.22E+08	1.11E+09	5.71E+08	6.96E+08	1.22E+09	6.31E+08	4.40E+08		
			3.52E+08	4.19E+08	3.99E+08	3.96E+08	5.24E+08	4.78E+08	3.73E+08					
	**1**	10 (5/5)	4.61E+08	8.47E+08	4.69E+08	3.83E+08	4.78E+08	2.85E+08	2.38E+08	1.56E+08	4.08E+08	2.15E+08	3.51E+08 ± 5.90E+07	4.37E+08 ± 1.14E+08
	**2**	10 (5/5)	5.22E+08	1.30E+09	3.24E+08	4.21E+08	7.77E+08	5.54E+08	9.64E+08	2.64E+08	4.73E+08	6.77E+08	4.83E+08 ± 5.84E+07	7.71E+08 ± 1.76E+08
	**3**	10 (1/9)	5.21E+08	6.00E+08	8.31E+08	8.59E+08	3.52E+08	3.08E+08	3.99E+08	9.06E+08	5.96E+08	4.75E+08	5.21E+08	5.92E+08 ± 7.60E+07
	**4**	10 (6/4)	8.55E+08	5.67E+08	7.56E+08	7.29E+08	8.96E+08	7.80E+08	6.07E+08	5.76E+08	6.19E+08	6.72E+08	7.64E+08 ± 4.70E+07	6.19E+8 ± 1.99E+07
	**5**	10 (4/6)	2.28E+09	1.33E+09	2.91E+09	1.27E+09	1.88E+09	1.29E+09	5.07E+08	2.42E+08	2.39E+08	3.55E+08	1.19E+09 ± 6.17E+08	1.26E+09 ± 3.18E+08
	**6**	10 (2/8)	2.61E+09	4.03E+08	1.36E+09	1.88E+09	1.23E+09	9.19E+08	1.11E+09	9.36E+08	7.15E+08	6.50E+08	1.14E+09 ± 2.21E+08	1.19E+09 ± 2.57E+08
	**7**	10 (4/6)	7.49E+08	1.37E+09	7.63E+08	6.92E+08	7.42E+08	1.22E+09	1.53E+09	1.15E+09	2.81E+08	6.63E+08	8.48E+08 ± 2.16E+08	9.61E+08 ± 1.68E+08
	**8**	10 (3/7)	5.60E+08	9.10E+08	1.21E+09	7.41E+08	9.34E+08	1.28E+09	5.52E+08	3.23E+08	3.73E+08	8.32E+08	7.13E+08 ± 1.72E+08	7.96E+08 ± 1.41E+08
	**9**	10 (2/8)	1.63E+09	1.07E+09	1.44E+09	1.28E+09	1.15E+09	1.52E+09	2.00E+09	4.78E+08	8.50E+08	4.15E+08	1.18E+09 ± 1.05E+08	1.19E+09 ± 2.00E+08
												**Mean *Cf* 18S Count**	**7.85E+08 ± 7.34E+07**	**8.58E+08 ± 5.64E+07**
